# New Strategies in the New Millennium: Servant Leadership As Enhancer of Service Climate and Customer Service Performance

**DOI:** 10.3389/fpsyg.2017.00786

**Published:** 2017-05-16

**Authors:** Jorge Linuesa-Langreo, Pablo Ruiz-Palomino, Dioni Elche-Hortelano

**Affiliations:** Department of Business Administration Department, University of Castilla-La ManchaCuenca, Spain

**Keywords:** social-aware customers, servant leadership, service climate, customer service performance, Marketing 3.0

## Abstract

In a world in which customers are increasingly looking for solutions to their own concerns on how to make a better globalized world, new organizational strategies are emerging to approach the customer in the current third millennium. Servant leadership, which involves putting employees’ needs first and serving the broader society, is emerging as a new strategic mechanism to approach the customer in line with the new social values-driven Marketing 3.0 era. Yet research has ignored the role and the various mechanisms servant leadership might utilize to improve customer service performance of their service units. Spanning 185 hotels located in Spain, a sample of 247 service units –in close contact with customers– was used to investigate whether servant leadership enhances customer service performance through shaping a service climate within the service unit. Results revealed that service climate mediates the positive influence of servant leadership on customer service performance. Managers can use these findings to note the value of leading the service unit in a servant friendly direction, which is better aligned with the new aspirations of customers today.

## Introduction

Recent marketing literature has begun to nurture from a new, very incipient perspective, the social values-driven Marketing 3.0 paradigm (Kotler et al., 2010), which proffers the idea that customers are increasingly seeking solutions to their own concerns and are interested in building a better world. Such understanding involves purchase decisions on the basis of fulfilling social and ethical values (e.g., social justice, human welfare, environmental sustainability; Shaw et al., 2005; Hollenbeck and Zinkhan, 2010). In other words, in this new millennium, driven by the Marketing 3.0. paradigm, which entails a more human-centric perspective, customers look to products and services to meet their own needs in parallel with fulfilling spiritual, social and moral values (Kotler et al., 2010). The extent to which a product or service provides freedom of choice, independence as well as benevolence, social justice, equality and environmental responsibility is becoming more and more crucial for customers when making purchase choices (Martínez-Cañas et al., 2016), especially in a developed world, where consumption appears to have become an end in itself, through which customers find a voice to promote a better society (Vrontis and Thrassou, 2007). Furthermore, customers are increasingly showing concerns about the effects of their purchase choices not only for themselves, but also for broader society (Harrison, 2005; González and Fernández, 2016), which represents a strong embracement of transcendent motives in their actions. In other words, in addition to making purchase decisions with an eye on external benefits gained (i.e., extrinsic motivation) or the pleasure acquired from the purchase decision itself (i.e., intrinsic motivation), customers are more and more concerned about whether their purchase decisions contribute to solving the problems of someone else (i.e., transcendent motives). In effect, in the current millennium, customers are more and more worried about whether others, both known or unknown, meet human good, such as truth, beauty, work, friendship, life, and dignity, such that their purchase decisions’ impact on others is carefully calculated (Martínez-Cañas et al., 2016).

In accordance with the described scenario, in which customers are demanding that businesses act in a socially responsible manner (Vrontis and Thrassou, 2007), it is of no surprise that managers are beginning to think of new customer-focused strategies to engage with the modern customer today (González and Fernández, 2016). One of these interesting strategies to appeal to the customer in the current social values-driven Marketing 3.0 era is the development of servant leadership in service units –work units which are in close contact with customers–. While leadership is deemed a central aspect to orientate service units’ mission and values toward a greater involvement and co-responsibility with broader society, servant leadership is the unique leadership approach which, as captured by its name, focuses on serving others (Liden et al., 2014), including the least privileged in society (Greenleaf, 1977). Servant leadership’s concerns extend beyond the organization itself to meet the well-being of followers, customers, other stakeholders, and society in a wider sense (Greenleaf, 1977; Barbuto and Wheeler, 2006). Their profound responsibility to serve others and contribute to the larger society is central in undertaking such leadership approach (Liden et al., 2014). In addition it purports to show followers how to fulfill the business mission of serving broader society, (Graham, 1991). Indeed, according to Greenleaf (1977), the first to coin the term after reading the Journey to the East by Herman Hesse, one of the central tenets of the servant leadership approach is that serving others entails encouraging others to do the same, so that they become servant leaders.

It is of no surprise then that this leadership approach results in an appropriate strategy to improve customer service (Brownell, 2010; Wu et al., 2013). When servant leadership is present in service units, employees of such work units are more likely to provide genuine care to customers and, in turn, authentic, high-quality customer service (Brownell, 2010). This process is possible because it is highly characteristic of servant leaders to fuel a cycle of service within their service units; servant leaders are role-models of servant behavior which, in turn, is mirrored by followers (Hunter et al., 2013). Therefore, under the influence of servant leaders a service climate, i.e., workers’ perception that internal practices, procedures, and behaviors support the provision of quality service– is likely to emerge. Furthermore, by shaping service climates, servant leaders should contribute to the enhancement of customer service performance, i.e., the workers’ proficiency in undertaking the core parts of their service role to provide high-quality customer service. This finds support in service-linkage research (Wiley, 1996; Pugh et al., 2002; Schneider et al., 2005), which is concerned with finding the links between employees and customers in service firms -where the boundaries between both agents are fairly permeable-. According to this perspective there must be specific drivers which link employees’ perceptions of various inter-organizational practices to customer perceptions, and some studies have argued that service climate is the bridge, the missing link between what happens inside –procedures, practices–, and what happens outside –customers’ perceptions– (Schneider et al., 2002; Hong et al., 2013). In fact, prior research has linked service climate to perceptions of service quality (Gracia et al., 2010), and service performance (Liao and Chuang, 2007).

It seems then that servant leaders in the new millennium, which depicts a scenario where customers are more concerned about how to contribute to build a better world, might play a role in improving customer service performance; however, such influence might occur by fostering and shaping service climates within their service units. To the best of our knowledge, this particular point has not yet been addressed in existing research. Therefore, this study pursues two main objectives. First, we will investigate whether servant leadership might be a suitable leadership strategy to improve customer service performance, as a measure which captures service units’ emphasis on service quality. Second, and more importantly, we examine the mediating effect of service climate between servant leadership and customer service performance. These relationships will be analyzed at the work unit level via spanning service units. The work unit, rather than the individual, is the building block of organizations, today (West and Markiewicz, 2004) and permits managers to work closely with followers on a daily basis. Service units in service, tourism firms, i.e., hotels, represent suitable units of analysis for uncovering the influence of servant leadership on service climate and service customer performance.

## Theory and Hypotheses Development

### The Nature of Servant Leadership

With its strong, unique focus on serving others (Wu et al., 2013; Liden et al., 2014), servant leadership offers unique aspects which can enhance the quality of service provided to customers. Greenleaf (1977, p. 27) coined the concept of servant leadership, though he failed to give a formal definition, and described the phenomenon of servant leadership as:

The servant-leader is servant first. It begins with the natural feeling that one wants to serve, to serve first. Then conscious choice brings one to aspire to lead. The difference manifests itself in the care taken by the servant-first to make sure that other people’s highest priority needs are being served. The best test, and difficult to administer, is this: Do those served grow as persons? Do they, while being served, become healthier, wiser, freer, more autonomous, more likely themselves to become servants? And, what is the effect on the least privileged in society? Will they benefit or at least not be further deprived?

Such description clarifies two core aspects of the servant leadership strategy. First, servant leadership extend their service approach to the various stakeholders, including employees, customers, and society in general (Graham, 1991; Wu et al., 2013); these leaders even raise strong concerns focused on improving the well-being of the least privileged in society. As such, because the servant leader’s area of concern extends beyond the business organization and includes the broader social environment (Brownell, 2010), these leaders demonstrate a high level of social responsibility for the well-being of society in general (Reinke, 2004). Second, servant leadership prioritizes the fulfillment of others’ needs above their own personal needs (Greenleaf, 1977), and inspire followers to develop intelligently, be creative and self-manage in order to serve others (Liden et al., 2008). As a result, because servant leaders practice this “service” mindset in all aspects of their lives (Liden et al., 2008), the principle of serving others above serving oneself is unlikely not to radiate out toward followers’ mindsets, attitudes, and behaviors. In effect, servant leaders encourage their followers to develop servant behaviors that will benefit all stakeholders (Liden et al., 2008; Sendjaya et al., 2008), thus ensuring that the strategies and decisions they opt for will offer a positive legacy to society (Barbuto and Wheeler, 2006). Because servant leaders are constantly searching out benefits to society (Searle and Barbuto, 2011), they are likely to inspire related servant attitudes in their followers to do their best to the benefit of all stakeholders, including customers. Thus, by developing a deep level of identification with the behavior carried out by their servant leaders (Zhang et al., 2012), employees should take personal responsibility for providing assistance and worth to customers, and thus provide services which are authentic and of superior quality (Brownell, 2010).

### Servant Leaders and Customer Service Performance

One of the key characteristics that sets service companies apart from those which produce goods is the simultaneous nature of service production and consumption, which, in many cases, results in consumer participation in the co-creation of the service (Bowen and Schneider, 1988). As such, the experience provided to the consumer upon receiving services is as important as, or even more important than, the product offered to the consumer (Bowen and Waldman, 1999). This has led to a paradigm shift for service companies when defining (customer service) performance, moving away from a focus on behavior evaluation to achieve organizational objectives (Campbell et al., 1993), toward a greater focus on behavior developed by the worker himself/herself, which is geared toward serving and helping the customer with the goal of providing high-quality service (Liao and Chuang, 2004).

This change of perspective means that the worker maintains direct and ongoing contact with the customer, thus increasing uncertainty as to how to interact with the customer, as in many cases the customer demands an immediate solution (Skaggs and Galli-Debicella, 2012). In these circumstances, the development of leaders who offer their workers both guidance and common sense, and who consistently cover all of his workers’ needs, is vital for improving workers’ performance (McGrath, 1962; Morgeson et al., 2010). As such, given that servant leadership develops social responsibility when serving both workers and customers (Mahembe and Engelbrecht, 2014), servant leadership in managerial roles appears to be a key element to generate a higher level of customer service performance (Chen et al., 2015). In effect, servant leaders possess impressive conceptual abilities for offering workers direction, support and clarity in solving day-to-day problems (van Dierendonck, 2011). These aspects help workers cultivate a precise understanding of their changing environment and develop individual and group skills (Hu and Liden, 2011). Some of these skills are, for example, more creative performance (Neubert et al., 2008), independence and self-confidence (Liden et al., 2008), which facilitates behavior which is spontaneous and useful in meeting customer demands without needing any type of supervision (Chen et al., 2015).

The positive relationship between servant leadership and customer service performance can be explained using the social exchange theory (SET, Blau, 1964; Cropanzano and Mitchell, 2005). SET theory indicates that social relationships are based on norms of reciprocity (Gouldner, 1960), where people look to maintain psychological balance in their social interactions, returning “favors” to those who have demonstrated proactive and positive tendencies toward them. Accordingly, when servant leaders in service units put their workers first, and display a service attitude and sincere concern for covering workers’ needs of personal and professional growth (Greenleaf, 1977), they create a psychological imbalance in workers’ relationships with these leaders. In a bid to benefit these leaders, workers might engage in service behaviors, directed to benefit the service unit, by superbly attending, for example, to customers’ needs. These behaviors, might also be impregnated with servant leaders’ strong emphasis in meeting the well-being of others, including customers and broader society, as these workers should become servants to an even greater extent according to Greenleaf (1977). As such, the display of such behaviors in encounters with customers should result in high-quality services, particularly in the present environment, in which customers are more and more needful of signals showing that the products and services they consume contribute to building a better society. Various studies support this relationship. While Netemeyer et al. (2005) recognize the better the leader–worker relationship, the stronger the impact is on the relationships maintained between workers and customers, other studies confirm a positive relationship between servant leaders and workers, which, in turn, translates into better worker–customer relationships and higher quality of service provided to customers (Jaramillo et al., 2009; Chen et al., 2015; Ling et al., 2016). Formally:

H1: *Servant leadership is directly, positively related to customer service performance*.

### Servant Leaders and Service Climate

Leadership is one of the most important factors in the process of climatic formation (Kozlowski and Doherty, 1989). Supervisors who model their leadership approach to all of their workers represent significant influences in forming the climate and providing it with content (Mayer et al., 2007). As such, servant leaders, by maintaining a service attitude oriented to meet both workers and customers’ needs (Wu et al., 2013), are active agents in forming a service climate within the work units to which these leaders belong (Walumbwa et al., 2010). In effect, service climate, defined as “employee perceptions of the practices, procedures, and behaviors that get rewarded, supported, and expected with regard to customer service and customer service quality” (Schneider et al., 1998, p. 151), is likely to emerge in service units which are led by servant leaders, as explained by social learning theory (SLT, Bandura, 1977, 1986).

Social learning theory contends that individuals learn the appropriate behavior by observing and emulating values, attitudes and behaviors of attractive, credible role models (Bandura, 1977, 1986). Managers become these influential referents (Mayer et al., 2012) because they embody proximity, frequent social interaction, and formal authority (Merton, 1957; Sutherland and Cressey, 1970), which makes it easier for them to garner attention and convey attractive information. As such, workers, through observing supervisors’ behaviors in a day-to-day work setting, are likely to engage in imitative behaviors (Hall and Lord, 1995), which can be further intensified whenever workers perceive their leaders to be in possession of qualities they consider to be attractive (Neubert et al., 2008; Mayer et al., 2012).

Servant leaders capture such attractiveness as they offer guidance and direction to workers and, by being humble, loving, empathetic, and servant (Sun, 2013), manifests sincere concern for satisfying the needs of both workers and customers (Wu et al., 2013). Imitative behavioral trends are highly probable in workers who are led by servant leaders, who will manifest behaviors that are similar to those of their leaders (Hunter et al., 2013), behaviors which are oriented to serve broader society by developing people committed, in turn, to serve society (Sims, 2005). This service-oriented behavior, developed by workers, results in a phenomenon of contagion (Bono and Ilies, 2006) among all members of the service unit which inspires a continuing service cycle (Hunter et al., 2013), a service-oriented culture (Liden et al., 2014) and, overall, a greater service climate (Walumbwa et al., 2010). The result is the creation of a work environment where members of the service unit share social behavioral norms, aimed first and foremost at offering high-quality service (Schneider et al., 2002). Accordingly, we propose:

H2: *Servant leadership is directly, positively related to service climate*.

### Service Climate and Customer Service Performance

Organizational theorists emphasize the importance of the organizational climate in determining employees’ attitudes and behaviors (Schneider et al., 2013). In accordance with the social information processing theory (SIP, Salancik and Pfeffer, 1978), employees collect the various messages released by their work units and utilize this information for decision making issues. Employees usually tend to adapt their feelings, attitudes, and behaviors according to what they perceive in their immediate work environment (Van Dyne et al., 1995). Therefore, the organizational climate, understood as the shared perceptions regarding policies, procedures, and practices (Schneider, 1990) which signal how things ought to be done and what behaviors are proper in the work environment (Liao and Chuang, 2007), represents an important influence on employees’ performance (Schneider and Barbera, 2014). In fact, depending on the specific dimension the organizational climate emphasizes, a number of studies have revealed its significant influence on specific behavioral outcomes regarding areas such as safety (i.e., Zohar and Luria, 2004), ethics (i.e., Deshpande, 1996), innovation (Anderson and West, 1998) or service (i.e., Schneider et al., 2009). This is because such specific climates represent the best source of cues to interpret events, undertake proper attitudes, and understand behavioral expectations concerning the different areas or dimensions emphasized.

In the particular case of service climate, it helps employees internalize that excellent service is expected, desired, and rewarded; it also represents a strong motivational force to deliver the best service in day-to-day activities (Liao and Chuang, 2007; Liao et al., 2009). Such perception is important in the service context, where services are produced and delivered in real time by unit employees (Ehrhart et al., 2011); in such contexts, the more customers perceive service quality is central for employees, the better their service experience (Schneider and White, 2004). This is not surprising as this specific climate emphasizes service quality to a great extent, so as a result, it should have a direct impact on service outcomes (Schneider, 1990) such as customer service performance. Indeed, employees highly engaged and sharing common perceptions about providing good quality of service to customers should perform well with customers (Salanova et al., 2005). This should occur because of social learning mechanisms (Bandura, 1977, 1986) as well as because perceptions that a high value for service is the tone, which should provide meaning to work and make employees enjoy their jobs to a greater extent (Hong et al., 2013). Earlier empirical research has consistently revealed that service climate enhances customer service performance (Borucki and Burke, 1999; Liao and Chuang, 2004, 2007; Salanova et al., 2005; Ehrhart et al., 2011; Hong et al., 2013). Thus, we predict:

H3: *Service climate is directly, positively related to customer service performance*.

### Servant Leaders-Customer Service Performance: The Mediation of Service Climate

According to linkage research (i.e., Wiley, 1996; Schneider et al., 1998; Wiley and Brooks, 2000; Pugh et al., 2002), there are internal elements of the work environment which can be strongly linked to critical external performance outcomes. Service climate is one of these internal elements which recent research has identified as the bridge between the work environment -as perceived by employees- and critical, external performance success factors oriented to the customer (e.g., customer service performance, Hong et al., 2013). This is important because it permits managers to apply an indirect approach, by focusing on more easily manageable internal aspects (e.g., leadership), to encourage customer service-minded behavior (Solnet, 2007), which can be conducive of better customer service perfomance.

Looking into internal elements, servant leadership implies an environmental stimulus (Hu and Liden, 2011) which is built upon service values based on genuine concern for and loving care of others (van Dierendonck, 2011; Hunter et al., 2013). Such stimulus should germinate, grow and propagate within the collective, because drawing on SLT (Bandura, 1977, 1986) workers feel attracted to imitate such attractive leaders by engaging in similar servant behaviors (Liden et al., 2014). Hence, servant leadership within service units should be associated with the shared perception that interpersonal relationships rest upon such service values, which should help shape a climate fostering helpful behavior oriented to offer high quality service (i.e., Walumbwa et al., 2010). Servant leaders foster climates which send clear messages that egotistical behavior is not tolerated (Liden et al., 2014), and service spirit is strongly encouraged (Liden et al., 2008).

Looking at external performance outcomes, various studies have revealed clear positive effects of service climate on customers (Bowen and Schneider, 2014), such as, for example, customer service (e.g., Schneider and Bowen, 1985), customer satisfaction (e.g., Schneider et al., 1996), and customer loyalty (e.g., Salanova et al., 2005). This is because in scenarios where service climate is perceived, workers share the understanding that the behavioral norms and expectations are to prioritize the needs of others (Liden et al., 2014), specifically, customers, which encourages employees’ strong engagement in high-quality service behavior directed to the customer (Liao and Chuang, 2007). Such a service climate is ignited through a spillover process spreading service attitudes and behaviors which should be noted in employee–customer interactions. With customers perceiving employees to be warm, in a good mood, and willing to dedicate time to understand their needs, customers get a good feeling about the service received. Such good feelings would also be nurtured as long as these influenced servant workers show strong concern for building a better society –which is an increasing concern of customers, today (Vrontis and Thrassou, 2007)-. In other words, by observing workers who behave in this way, customers should enjoy such an awesome experience that they should feel that a high quality service has been received (Hong et al., 2013).

Overall, by combining both internal and external perspectives, we contend that because servant leaders enhance service climate within their service units (Walumbwa et al., 2010), these leaders improve the quality of service that workers offer to customers (Ling et al., 2016). In other words, by drawing on the broad existing body of linkage research (Wiley, 1996; Schneider et al., 1998; Wiley and Brooks, 2000; Pugh et al., 2002), we contend that service climate is the bridge between servant leadership and customer service performance. Thus, we propose:

H4: *Service climate mediates the relationship between servant leadership and customer service performance*.

**FIGURE 1:**
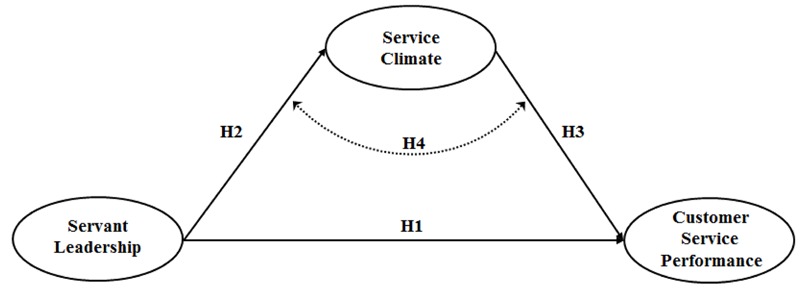
**Hypothesized model**.

This last hypothesis combined with the previous ones make it possible to summarize our research model as displayed in **Figure 1**.

## Method

### Sample and Procedure

In order to test these relationships, we conducted surveys to gather data in Spain’s hospitality industry, which is likely to attract managers who are servant leaders. Customer service of the utmost quality is key for success of companies in this sector, so servant leadership strategies could make an important difference and be common in these circles (Wu et al., 2013).

In an effort to minimize common method bias (CMB), and social desirability bias (SDB), we selected the most fitting participants for all our study constructs (Podsakoff et al., 2003). Firstly, workers were selected to measure the extent to which their supervisors can be portrayed as using a servant leadership strategy. Secondly, service climate was measured by using both workers’ ratings and supervisors’ ratings, as it allows us to minimize the same-source bias problem (Ostroff et al., 2002) and thus have a more objective indicator of the phenomena. Finally, customer service performance was assessed by the hotel general manager, not by service units’ supervisors who might give biases responses. Accordingly, we designed three different questionnaires for each target respondent (i.e., hotel general manager, service unit supervisor, service unit worker). We pilot tested each questionnaire with a convenience sample of 3 general managers, 10 supervisors, and 25 workers in 3 hotels, respectively, which confirmed the clarity, comprehension, readability, and suitability of the items included. Surveys’ cover letters for each target respondent indicated absolute anonymity, and noted that only aggregated data would be utilized for research purposes.

Once consent was gained from the general managers at 185 hotels, each located in a distinct Spanish historical site, data from 247 service units (three members per unit at a minimum) which were in close contact with customers (e.g., reception desk, restaurant) could be gathered. Both supervisor and workers’ responses were collected per each service unit; in total 840 responses were received –the response rate was high, around 77%-. As to collection of data concerning each unit’s customer service performance, hotel general managers were also surveyed; in total 185 responses were obtained. Data were collected at specific sites in each establishment, which helped us ensure that each survey was paired with its corresponding service unit and hotel.

As further countermeasures for the CMB and SDB issues, the design of the survey was based on recommendations raised by Podsakoff et al. (2003) and Conway and Lance (2010). For example, the survey’s cover letter highlighted the fact that there were no correct or incorrect answers, thanked participants in advance for being honest, and noted that all responses would remain anonymous. Participants did not have to share their names, job titles or employers’ names in the survey. Furthermore, the cover letter clearly stated that the results were for academic purposes only, thus reducing SDB (Nancarrow et al., 2001). Lastly, when designing the survey (Podsakoff et al., 2003), we worked to ensure a psychological separation between predictors and outcome variables, to keep them from seeming related. We also used distractor elements and utilized items that were simple, focused and concise.

### Measures

The survey was conducted in Spanish. Brislin’s (1980) back-translation procedure was conducted to our mediator and independent variable, and no meaningful differences between the two translations from and to English were noticed. An exhaustive analysis, according to MacKenzie et al.’s (2005) criteria, showed that all our measures contained highly correlated indicators; in other words, our survey included reflective measures in all cases.

#### Servant Leadership

Service unit workers used Ehrhart’s (2004) reliable 14-item scale to rate servant leadership of their service unit supervisors. The scale used a seven-point response format (1 = “strongly disagree,” 7 = “strongly agree”). Sample items were, “My supervisor spends the time to form quality relationships with service unit employees” and “My supervisor emphasizes the importance of giving back to the community.” Because we were interested in overall patterns of servant leadership behavior within the service unit, we averaged employees’ ratings within each service unit. To confirm that this aggregation of individual scores to the unit level was appropriate, we calculated the within-service unit agreement score (rwg, James et al., 1984) and two intraclass correlations: ICC(1), or the proportion of variance in ratings due to service unit membership, and ICC(2), or the reliability of service unit mean differences (Bliese, 2000). The average rwg value was 0.83, and the ICC(1) and ICC(2), were, respectively, 0.65 and 0.86, which met acceptable cutoffs (Bliese, 1998). In addition, we performed a one-way analysis of variance (ANOVA), which indicated significant differences across the service units in the average scores (*F* = 11.07, *p* < 0.01). Therefore, we consider the aggregation justified (Bliese, 1998).

#### Service Climate

All service unit members, including both workers and supervisors, completed on a seven-point response format (1 = “strongly disagree,” 7 = “strongly agree”), Carrasco et al.’s (2012) 4-item service climate scale, which is an adaption to the Spanish context of the Schneider et al.’s (1998) global service climate scale. Sample items were, “Employees in our service unit have knowledge of the job and the skills to deliver superior quality work and service,” and “The overall quality of service provided by our service unit to customers is excellent.” The ANOVA indicated significant differences (*F* = 5.48, *p* < 0.01), the median rwg value was 0.83, and the ICC(1) and ICC(2) score were 0.27 and 0.56, respectively; so within-unit agreement and between-unit differentiation supported the aggregation of respondents’ scores to the service unit level.

#### Customer Service Performance

Hotel general managers were asked to rate the performance of service units surveyed compared to the average of other work units in the hotel. We decided to ask hotel general managers instead of service units’ supervisors because general managers should offer more accurate, far less biased responses. Specifically, hotel managers had to respond on a 7-point response format (“very poor,” 1, to “excellent,” 5) to four items adapted from Oh et al. (2004) to measure the extent to which the service unit in question provided high quality service to the customer. Sample items include “service unit’s quality of work” and “service unit’s overall performance.”

#### Control Variables

In the present study, we introduced two control variables. We included *service unit education* to control for confounding effects because the educational level has been recognized in the past to influence helping behavior (Van Dyne and LePine, 1998) and successful service performance (Davidoff, 1994). *Service unit education* was measured by the average education level of participants in each service unit surveyed; individual responses on a six-point format response (1 = primary studies; 2 = secondary studies; 3 = lower level professional education; 4 = higher level professional education; 5 = bachelor degree; 6 = postgraduate degree) were averaged within each service unit. Also, we controlled for *service unit size* effects because it can affect group dynamics (e.g., interpersonal contacts, synergies) and performance, positively (Brewer and Kramer, 1986; Smith et al., 1994). We measured service *unit size* by the number of workers who participated in each service unit we surveyed.

### Data Analysis

We utilized partial least squares via Smart PLS 3.2.6 (Ringle et al., 2015) to test our hypotheses. Such an impressive and potent statistical procedure (Chin et al., 2003) makes the causal analyses of complex situations possible (Henseler et al., 2009). As a structural equation modeling (SEM) approach, it is also suitable for testing mediation hypotheses (James et al., 2006). Also, PLS does not make it necessary to demand assumptions concerning the distribution of the variables (Henseler et al., 2009). As recommended, our PLS analysis used 5,000 subsamples to generate standard errors and bootstrap *t*-statistics with n-1 degrees of freedom (where *n* is the number of subsamples) to evaluate statistical significance of path coefficients (Henseler et al., 2009).

We tested the hypotheses at the unit level of analysis, using a sample of 247 service units (*n* = 247). To run our SEM model at the unit level, our servant leadership variable was formed by ratings provided by service unit workers which were averaged to yield service unit-level scores. Likewise, our service climate variable was constructed using ratings provided by the supervisor and workers of each service unit, that were averaged together to obtain unit-level scores. Finally, our criterion variable, customer service performance, was created based on ratings provided by the general manager of the hotel to which each service unit belonged to.

#### Measurement Model

By following the recommendations of Conway and Lance (2010), we present information related to good reliability and validity for our reflective measures as an additional test to show that CMB is not an issue in our study. **Table 1** shows evidence of individual and construct reliability, and convergent validity, while **Tables 2, 3** offer good discriminant validity for all our measures. In addition, **Table 2** shows the correlations across the variables.

**Table 1:** Item loadings, construct reliability and convergent validity.

Construct	Item	λ	Construct reliability		AVE
			α	ρ	
**SL**			0.98	0.98	0.81
	(1) My supervisor spends the time to form quality relationships with work unit employees	0.91			
	(2) My supervisor creates a sense of community among work unit employees	0.91			
	(3) My supervisor’s decisions are influenced by work unit employees’ input	0.91			
	(4) My supervisor tries to reach consensus among work unit employees on important decisions	0.92			
	(5) My supervisor is sensitive to work unit employees’ responsibilities on important decisions	0.88			
	(6) My supervisor makes the personal development of work unit employees a priority	0.92			
	(7) My supervisor holds work unit employees to high ethical standards	0.93			
	(8) My supervisor does what she or he promises to do	0.89			
	(9) My supervisor balances concern for day-to-day details with projections for the future	0.93			
	(10) My supervisor displays wide-ranging knowledge and interest in finding solutions to work problems	0.89			
	(11) My supervisor makes me feel like I work with him/her, not for him/her	0.92			
	(12) My supervisor works hard at finding ways to help others be the best they can be	0.93			
	(13) My supervisor encourage work unit employees to be involved in community service and volunteer activities outside of work	0.82			
	(14) My supervisor emphasizes the importance of giving back to the community	0.82			
**SC**			0.92	0.95	0.82
	(1) Employees in our work unit have knowledge of the job and the skills to deliver superior quality work and service	0.94			
	(2) Employees receive recognition and rewards for the delivery of superior work and service	0.92			
	(3) The overall quality of service provided by our work unit to customers is excellent	0.93			
	(4) Employees are provided with tools, technology, other resources to support the delivery of quality work and service	0.82			
**CSP**			0.86	0.91	0.71
	This work unit’s quality of work	0.86			
	This work unit’s initiative	0.79			
	This work unit’s ability to complete work on time	0.82			
	This work unit’s overall performance	0.90			

*All *t*-values of the individual loadings are significant at *p* < 0.001 or better. SL = servant leadership; SC = service climate; CSP = customer service performance; **λ =** item loading; α = cronbach’s alpha; ρ = composite reliability; VIF = variance inflation factor. AVE = average variance extracted.*

**Table 2:** Descriptive statistics, correlation matrix and discriminant validity (√AVE in bold).

	Mean	*SD*	SL	SC	CSP	SUE	SUS
SL	5.06	1.18	**0.90**	0.68 [0.54;0.78]	*0.20 [0.06;0.39]*	*0.03 [0.01;0.04]*	*0.07 [0.02;0.20]*
SC	5.43	0.82	0.65	**0.90**	*0.35 [0.17;0.51]*	*0.06 [0.01;0.19]*	*0.14 [0.05;0.26]*
CSP	5.79	0.77	0.18	0.32	**0.84**	*0.13 [0.02;0.28]*	*0.08 [0.01;0.17]*
SUE	3.90	1.07	0.01	0.06	0.12	**n.a.**	*0.03 [0.00;0.11]*
SUS	8.53	4.92	0.07	0.14	0.07	0.03	**n.a.**

*Bold values on the diagonal are square roots of AVE (variance shared between the constructs and their measures). Off-diagonal elements below the diagonal are correlations among the constructs, where correlations between 0.12 and 0.16 are significant at *p* < 0.05 (two-tailed), and correlations above 0.16 are significant at *p* < 0.01 (two-tailed). Because the square root of each reflective construct’s AVE is higher than its correlation with another construct, discriminant validity is established in light of the Fornell-Larcker criterion (Henseler et al., 2009). Off-diagonal elements above the diagonal are the heterotrait–monotrait ratio of correlations (HTMT), italicized values are the confidence intervals; because the HTMT value is always below 0.85, and bias and corrected confidence intervals at the 99% level of significance do not include 1, discriminant validity is supported (Henseler et al., 2015). SL, servant leadership; SC, service climate; CSP, customer service performance; SUE, service unit education; SUS, service unit size; SD, standard deviation.*

**Table 3:** Cross-loadings matrix for reflective constructs.

Items	Servant leadership	Service climate	CSP	Service unit education	Service unit size
SL1	**0.91**	0.57	0.18	0.00	0.09
SL2	**0.91**	0.60	0.20	0.00	0.10
SL3	**0.91**	0.61	0.21	0.08	0.05
SL4	**0.92**	0.65	0.19	0.04	0.10
SL5	**0.88**	0.59	0.17	0.04	0.08
SL6	**0.92**	0.59	0.17	0.01	0.05
SL7	**0.93**	0.60	0.17	-0.05	0.07
SL8	**0.89**	0.57	0.14	-0.01	0.09
SL9	**0.93**	0.60	0.15	0.02	0.03
SL10	**0.89**	0.57	0.15	0.00	0.08
SL11	**0.92**	0.61	0.19	0.00	0.09
SL12	**0.93**	0.59	0.19	-0.02	0.07
SL13	**0.82**	0.49	0.11	-0.08	0.02
SL14	**0.82**	0.51	0.10	-0.06	0.01
SC1	0.60	**0.94**	0.28	0.07	0.17
SC2	0.60	**0.92**	0.25	0.07	0.16
SC3	0.61	**0.93**	0.36	0.07	0.13
SC4	0.54	**0.82**	0.25	0.00	0.05
CSP1	0.12	0.27	**0.86**	0.07	0.07
CSP2	0.15	0.28	**0.79**	0.12	0.02
CSP3	0.15	0.25	**0.82**	0.09	0.08
CSP4	0.20	0.27	**0.90**	0.12	0.07
SUE	0.01	0.06	0.13	**1.00**	0.03
SUS	0.07	0.14	0.11	0.03	**1.00**

*Bold figures indicate that each item loaded highest on its associated construct, so all these constructs are conceptually distinct (Henseler et al., 2009). CSP, Customer.*

As seen in **Table 1**, all the individual items of the reflective variables, whose standardized loadings are far above the threshold of 0.70, are reliable (Henseler et al., 2009). In addition, the Cronbach’s alpha values and composite reliability values both point to good reliability and internal consistency for all our reflective constructs, with values that are above the desired threshold of 0.80, as required for basic research (Nunnally, 1978, Table 1). The convergent validity condition was also met, because the average variance extracted (AVE) values related to each reflective construct were far above 0.50 (Henseler et al., 2009, Table 1). Lastly, we looked at the divergent validity of our reflective measures using a variety of methods. On the construct level, the criterion of Fornell and Larcker (1981) was achieved to our satisfaction, given that the AVE for each construct was greater than the variance shared by each construct with the other latent variables (**Table 2**) (Henseler et al., 2009). In addition, the heterotrait–monotrait (HTMT) criterion backed this point up as the HTMT values among our study variables were all far lower than even the most conservative 0.85 cut-off (**Table 2**), hence verifying discriminant validity for each pair of constructs (Henseler et al., 2015). This issue was also upheld when the HTMT inference criterion was utilized, which tests the null hypothesis (H_0_: HTMT ≥ 1) against the alternative hypothesis (H_1_: HTMT < 1), concluding that HTMT values among our study variables are markedly different from 1, given that confidence intervals did not include this value (Henseler et al., 2015, Table 2). On an item level, we could also affirm that our reflective constructs were different, since the cross-loading matrix showed that all items loaded on their intended constructs more than on any other construct (**Table 3**) (Henseler et al., 2015). Overall, the discriminant validity of our study variables can be considered acceptable.

### Hypotheses Testing

**FIGURE 2:**
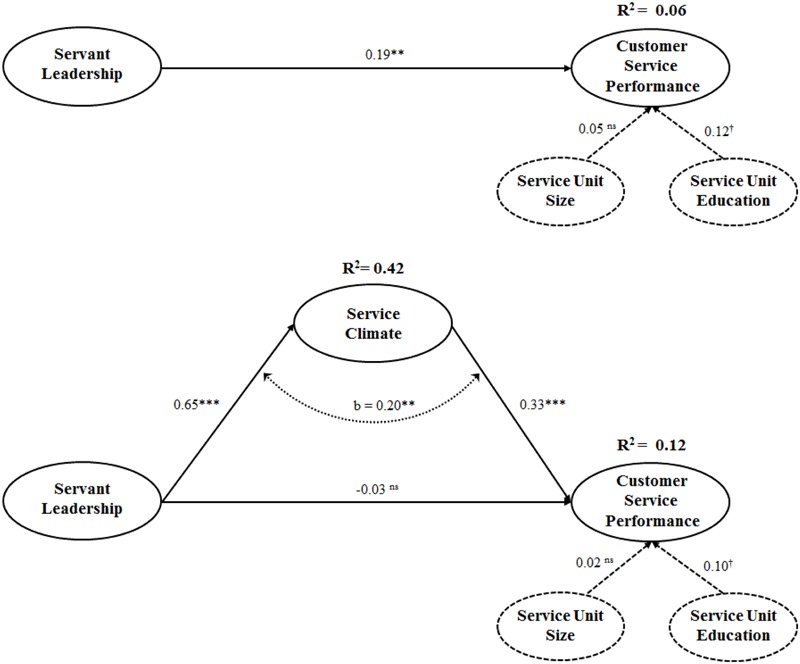
**The servant leadership-customer service performance relationship: The mediation of service climate.** Bootstrapping based on *n* = 5,000 sub samples, where a bootstrap *t*-statistic with *n* = 1 degrees of freedom is used (*n* is the number of subsamples). VIF values for the complete model range between 1.00 and 1.76, far below the 5.0 cut-off (Hair et al., 2017), so path coefficients do not suffer from multicollhiearity problems. ^†^*p*< 0.10 (two-tailed test); ^∗∗^*p*< 0.01 (one-tailed test); ^∗∗∗^*p*< 0.001 (one-tailed test); n.s. = not significant.

The variance associated with our control variables was practically non-existent as **Table 4** and **Figure 2** reveals. Only service unit level of education was significantly, positively related to service unit’s customer service performance (β = 0.10, *p* < 0.10), thus suggesting the importance of considering this aspect when configuring the workforce of service units. **Table 4** and **Figure 2** contain findings concerning our hypotheses, as well. Contrary to our expectations concerning H1, servant leadership was not directly related to customer service performance (β = -0.03, not significant, **Table 4** and **Figure 2**), so our H1 could not be supported. However, we found support for H2 and H3, because servant leadership related directly, positively to service climate (β = 0.65, *p* < 0.001, **Table 4** and **Figure 2**) and service climate was directly, positively related to customer service performance (β = 0.33, *p* < 0.001, **Table 4** and **Figure 2**). Thus, while servant leadership was not found to influence customer service performance directly, our findings reveal that servant leadership is an important antecedent of service climate, which, in turn, impacts customer service performance, in clear support of H2 and H3, respectively.

**Table 4:** Servant leadership–CSP relationship: direct, indirect, total effects, and variance explained.

Effects on dependent variables	Direct effects (*t-value*)	Indirect effects	Total effects	Variance explained	Effect sizes
**Service climate (*R*^2^ = 0.42)**				
Servant leadership	0.65^***^ (12.75)	–	0.65	0.42	Large
**CSP (*R*^2^ = 0.12)**			
Servant leadership	-0.03^ns^ (0.30)	*b* = 0.20^a^	0.20	0.00	n.a.
Service climate	0.33^***^ (3.70)	–	0.33	0.11	Medium
Service unit education	0.10^†^ (1.56)	–	0.10	0.01	Small
Service unit size	0.02^ns^ (0.41)		0.02	0.00	Null

For testing independent variables’ effects: ^***^p < 0.001 (one-tailed test): t_(4999_) = 3.092, ns: not significant. For testing control variables’ effects: ^†^p < 0.10 (two-tailed test): t_(4,999)_ = 1.645, ns: not significant. ^a^Based on a bootstrap test with 5,000 re-samples, the indirect effect = 0.20 is significant at p < 0.01. The bias-corrected and accelerated 95% confidence interval (CI) does not include the zero (CI lower level = 0.11; CI upper level = 0.31). Effect sizes of R^2^ ≥ 0.01, ≥ 0.09, and ≥0.25 are small, medium, and large, respectively (Cohen, 1988). CSP, Customer Service Performance.

To test H4, regarding the indirect effects of servant leadership on customer service performance, we adopted Preacher and Hayes’s (2004) approach. In a bootstrap test with 5,000 subsamples (Hayes, 2009; Preacher and Hayes, 2004), the indirect effect was significant (*b* = 0.20, *p* < 0.01), and zero was absent from the 99% bias-corrected and accelerated bootstrap confidence intervals (CI lower level = 0.11; CI upper level = 0.31). The evidence of significance of this indirect effect suggests that mediation exists (Preacher and Hayes, 2004) and provides the empirical basis to analyze the mediation effect (MacKinnon et al., 2002). For the mediation test, we used Tippins and Sohi’s (2003) four-criterion procedure –which includes Baron and Kenny’s (1986) criteria– but applies to SEM better because it compares an unmediated model with a mediated model to find significant differences (**Figure 2**) and test if these four statistical conditions are met. The first criterion was met because the mediated model accounted for more variance in consumer service performance than the unmediated model (**Table 5** and **Figure 2**). Also, in line with H2, servant leadership related positively, directly to service climate, which offered support for the second requirement for mediation. Likewise, our results confirmed the third condition, because service climate had a significant, positive, direct effect on customer service performance, which was also medium in size (*R*^2^ = 0.11; **Table 4**). Finally, according to the fourth condition, there was a significant positive effect of servant leadership on customer service performance in a model in which the mediator was excluded (β_Unmediated Model_ = 0.19, *p* < 0.01), but dropped to null when the mediator was added, implying full mediation (β_Mediated Model_ = -0.03, n.s.) (See **Figure 2**). In summary, although this mediation effect was small in size (*f*^2^ ≥ 0.02; **Table 5**) (Cohen, 1988), our results reveal that service climate mediates the relationship between servant leadership and customer service performance, prominently. Thus, the positive impact of servant leadership on customer service performance is not direct, but indirect, through enhancing service climate, in full support of H4.

**Table 5:** Mediation effect size of service climate.

Dependent variable	Variance explained			Mediation Strength *f*^2^)
	Direct model	Mediated model	Δ Variance explained	
Customer service performance	0.06	0.12	0.06	0.07 (small)

*f^*2*^ = (*R*^*2*^ included – *R*^*2*^ excluded)/(1–*R*^*2*^ included); effect sizes of *f*^*2*^ ≥ 0.02, ≥0.15, and ≥0.35 are small, medium, and large, respectively (Cohen, 1988).*

## Discussion and Conclusion

### Theoretical Contributions

In the service industry, the quality of employee–customer interactions is deemed a critical aspect to gain excellent customer service performance. Such encounters often represent the only contact customers have with the organization, so managers should manage these interactions properly (Solnet, 2007). However, although traditional control mechanisms can help manage such interactions (i.e., reward and punishment systems, incentives), it is not enough to control every conscious or unconscious word, gesture and attitude employees may show. With this study we provide new strategies far less based on control mechanisms as helpful in approaching customers properly, especially in the current times in which customers are increasingly social-aware. Specifically, we contributed to showing how the generation and development of servant leadership behaviors in managerial roles within service units leads to higher customer service performance, if only because servant leadership enhances service climate. It is important because it sends the clear message to managers that what they do in their day-to-day worklife, including attitudes, gestures, words, behaviors, matters to the point of enhancing service climate, and, in turn, customer service performance of their service units.

In this study we focused on the mediating role of service climate in this relationship as a broad body of prior research, known as linkage research (i.e., Wiley, 1996; Schneider et al., 1998; Wiley and Brooks, 2000; Pugh et al., 2002), has highlighted its significant role as the bridge which links employee perceptions of internal factors with important external criterion measures of outcomes such as quality of employee–customers interactions. The results confirmed the key role of service climate in linking servant leadership to customer–servicer performance. As we expected, service climate mediated the relationship between servant leadership of managers and the service unit’s customer service performance and did it in a complete way, as the direct effect of servant leadership disappeared when service climate was included. Such a finding is in line with and qualifies prior research (i.e., Salanova et al., 2005) as it puts on the table that one specific leadership strategy, i.e., servant leadership, which is gaining increased attention over the years (van Dierendonck, 2011), is powerful in enhancing service climate, through which customer service performance can be ultimately improved. Servant leadership is unique in capturing servanthood, genuine concern in the growth of others, including workers, customers and the least privileged in society; this leadership approach shows care for social order as well as compassion and justice (Sims, 2005), which fits the rationales behind the values-driven Marketing 3.0 paradigm (Kotler et al., 2010). Hence, our study contributes to existing literature by highlighting the critical personal aspects (i.e., servanthood) managers should exhibit in their attitudes, values and behaviors, to shape a service climate which truly has an outstanding impact on customer service performance, especially in this new millennium in which customers are increasingly social aware.

### Practical Implications

The results of the present study allow us to suggest several implications from a practical, managerial perspective. For example, managers can use the knowledge in this research to note how important the leadership strategy they show is, and which aspects should be emphasized to improve customer firm performance. Specifically, managers should exhibit genuine servant behaviors, including genuine interest in serving workers, customers and broader society. To this end, the strategic plan managers can implement is twofold. First, training programs focused on coaching managers in the area of the servant leadership philosophy could be useful, even though the interest in developing a servant leadership approach should come from the inner self (Ling et al., 2016). Indeed, servant attitudes and behaviors can be learnt, as well (Brownell, 2010), so by implementing training programs which enable more empathetic disposition and stronger concern about needs of others to be shown, managers could learn to develop servant leadership. Second, human resource managers should emphasize servant leadership traits when hiring new managers. Using personality tests involving specific items which evaluate personal aspects such as honesty, servanthood, stewardship or empathetic orientation (Hunter et al., 2013) could help find the right candidate for the position of manager.

In addition, managers should not ignore the important role of shaping a service climate within their service units to gain excellent customer service performance. Servant leadership, and its focus on serving others over and above oneself is principal in shaping such a service climate, and can involve, in turn, the design and implementation of a number of processes as described next. For example, servant managers should make sure that a human resources practices system oriented to both support workers in their day-to-day interactions with customers and provide these contact workers with the relevant knowledge and skills to succeed (by offering a high quality service) is properly implemented. Also, this system should serve: (a) to send clear information concerning standards of customer service to be provided, (b) to educate employees about how to perform in employee–customers encounters, properly, and (c) to design two-way communication channels which make managers realize problems and needs of employees in their daily tasks, and personal interactions with customers. Overall, managers should devote time and energy to serve contact workers, including providing due resources to approach customers, properly, so workers can share the idea that all the functioning of their specific service units focus on service quality, and thus emphasize a strong service climate.

### Limitations and Further Research

Our findings must be considered in light of some limitations. Some stem from our research design. One limitation is, for example, that because our investigation was designed in a cross-sectional manner, we cannot offer strong causal inferences, so future research should include longitudinal designs to address our causality inferences more precisely. Also, our study was conducted in the customer service–oriented hospitality industry of historical sites situated in a specific cultural context (i.e., Spain); hence, future studies interested in generalizing our findings to other industries and cultural contexts should design cross-cultural studies spanning various, distinct service industries. Furthermore, although we collected our data by three different ways (employees, supervisors, hotel general managers), which improves data reliability, and minimizes CMB to a great extent (Podsakoff et al., 2003), future research could include customers to evaluate the dependent variable in our investigation (customer service performance), as well as aspects such as customer satisfaction and quality of service. In this connection, future research interested in advancing our findings could also ask customers about their system of values, by utilizing scales such as the Rokeach’s (1973) or Schwartz’s (1994) values surveys; this could help test if the servant leadership-customer service performance is contingent upon customers who are more or less socially aware.

Another important limitation is that we examined service climate as a relevant mediating variable between servant leadership and customer service performance, but other mechanisms might explain this relationship, as well. For example, employee service unit identification has been recognized as having an important role in gaining good employee–customer interactions and customer satisfaction (Solnet, 2007). Chen et al. (2015), find that this variable and other social identity factors (i.e., service unit self-efficacy) might have to do something in this relationship. Future research could evaluate the mediating role of such social identity variables in our relationship, and test whether service climate increases customer service performance via igniting higher service unit self-efficacy and employee identification. Also, a multilevel analysis which evaluates, within our research model, the influential role of individual-level variables that are often enhanced by servant leadership (e.g., service attitude, altruistic behavior) represents an appealing area for future research.

Finally, we examined the influential role of servant leadership of supervisors within service units. This choice was made on the basis that this is the person with whom workers spend more time and interact most, which allowed us to investigate the effects of servant leadership on service climate and customer service performance, more accurately. However, some other studies have also demonstrated positive effects of general managers’ servant leadership on valuable organizational outcomes (Peterson et al., 2012; Huang et al., 2016). Thus, an interesting area of future research is to examine the trickle-down effect of servant leadership within the organization, and test the combined positive effects of servant leadership in the various hierarchical levels on both service climate and customer service performance, and at either the service or organization unit level.

In short, our investigation provides key insights about new strategies, i.e., servant leadership, to gain customer service performance in a new era in which customers are more concerned about building a better society. This research also reveals the mechanisms, i.e., service climate, by which servant leaders boost such customer service performance in service units, and provides a map for avenues of appealing, ongoing research.

## Author Contributions

JL-L participated in the data collection. JL-L, PR-P, and DE-H worked on the theoretical framework, methodology, results analysis and discussion in equal measure.

## Conflict of Interest Statement

The authors declare that the research was conducted in the absence of any commercial or financial relationships that could be construed as a potential conflict of interest.
